# Patient-reported supportive care needs among Asian American cancer patients

**DOI:** 10.1007/s00520-022-07338-2

**Published:** 2022-08-30

**Authors:** Katarina Wang, Carmen Ma, Feng Ming Li, Angeline Truong, Salma Shariff-Marco, Janet N. Chu, Debora L. Oh, Laura Allen, Mei-Chin Kuo, Ching Wong, Hoan Bui, Junlin Chen, Scarlett L. Gomez, Tung T. Nguyen, Janice Y. Tsoh

**Affiliations:** 1 Asian American Research Center On Health, University of California, San Francisco, CA USA; 2 Department of Epidemiology & Biostatistics, University of California, San Francisco, CA USA; 3 Helen Diller Family Comprehensive Cancer Center, University of California, San Francisco, CA USA; 4 Division of General Internal Medicine, University of California, San Francisco, CA USA; 5 Department of Psychiatry and Behavioral Sciences, University of California, San Francisco, CA USA

**Keywords:** Asian American, Cancer, Patient navigation, Multilingual, Cultural humility, Cultural competence, Supportive care needs

## Abstract

**Purpose:**

Cancer is the leading cause of death for Asian Americans. However, few studies have documented supportive care needs from the perspective of Asian American cancer patients. This study describes the needs reported by Asian American patients with colorectal, liver, or lung cancer over a 6-month period during their treatment.

**Methods:**

Participants were recruited through the Greater Bay Area Cancer Registry and from cancer care providers in San Francisco. Participants self-identified as Asian or Asian American; were age 21 or older; spoke English, Chinese, or Vietnamese; and had stage I–III colon, rectum, liver, or lung cancer. Participants were matched with a language concordant patient navigator who provided support during a 6-month period. Needs were assessed by surveys at baseline, 3, and 6 months.

**Results:**

Among 24 participants, 58% were 65 years or older, 42% did not complete high school, and 75% had limited English proficiency (LEP). At baseline, the most prevalent needs were cancer information (79%), nutrition and physical activity (67%), language assistance (54%), and daily living (50%). At the 3- and 6-month follow-up surveys, there was a higher reported need for mental health resources and healthcare access among participants.

**Conclusion:**

In this pilot study of Asian American cancer patients who predominantly had LEP, participants reported many needs, with cancer information and language assistance as the most prominent. The findings highlight the importance of culturally and linguistically appropriate patient navigators in addressing supportive care needs among cancer patients with LEP.

**Trial registration:**

ClinicalTrials.gov identifier: NCT03867916.

**Supplementary Information:**

The online version contains supplementary material available at 10.1007/s00520-022-07338-2.

## Introduction

In 2020, there were 24 million Asian Americans living in the USA [1]. This population increased by 36% in the past decade, making it the fastest growing racial group in the country [1, 2]. The Asian American population is highly heterogeneous in national origins and languages; 65% of Asian Americans speak a language other than English at home, and 34% do not speak English very well [3, 4]. Limited English proficiency (LEP) and health literacy lead to difficulties in communicating with healthcare providers, navigating the healthcare system, and understanding health information [5, 6].

Cancer is the leading cause of death among Asian Americans [6], with lung, colorectal, and liver cancers among the top five leading causes of Asian American cancer deaths [6, 7]. Despite the large number of Asian Americans that have cancer and the barriers of accessing care, there are few studies that have examined their supportive care needs, especially those who have LEP [8–11]. Cancer supportive care is defined as care given to prevent and manage the adverse effects of cancer and its treatment [12, 13]. Two cross-sectional surveys of Chinese American cancer survivors reported language barriers and stigmatization of cancer as common challenges to seeking medical attention and supportive care resources [7, 14]. Two systematic reviews on the supportive care needs of Chinese cancer patients across Asia, North America, and Australia reported that Chinese cancer patients have unmet needs in accessing information about cancer treatment, healthcare access, and finances [8, 9]. Few studies have included Asian American cancer patients undergoing treatment [9–11], and to our knowledge, no published studies assess Asian American cancer patients’ needs over time during their treatment.

The Patient Cancer OUtreach, Navigation, Technology and Support (Patient COUNTS) Study assesses the supportive care needs of Asian American cancer patients who speak Chinese, English, and Vietnamese and helps them access supportive care resources. Here, we describe patient-reported needs during their cancer treatment.

## Methods

### Study design

Patient COUNTS is a single-arm prospective cohort pilot study to design and test the feasibility and acceptability of a patient navigation program for Asian American patients newly diagnosed with colorectal, liver, or lung cancer (ClinicalTrials.gov identifier: NCT03867916). All research procedures were approved by the Institutional Review Board (IRB #18–25820) at the University of California, San Francisco (UCSF) and the state of California Committee for the Protection of Human Subjects (CPHS). Informed consent was obtained from all study participants. All consent and study materials were in English, Chinese, and Vietnamese.

A Patient Advisory Council (PAC), consisting of 15 Asian American members who were community leaders, cancer patients and their caregivers, and navigators, guided the development and implementation of the study. Languages spoken by PAC members included English, Cantonese, Mandarin, Taishanese, and Tagalog.

### Recruitment

The study period was from July 2019 to December 2020. Newly diagnosed patients were identified using an early case ascertainment (ECA) process from the Greater Bay Area Cancer Registry (GBACR), a population-based cancer registry that covers 9 counties in northern California and is part of the National Cancer Institute’s Surveillance, Epidemiology and End Results (SEER) program and the statewide California Cancer Registry [15]. To support research studies of recently diagnosed cancer patients, ECA makes cases available for eligible studies as soon as a record is created in the registry (about 6 months after diagnosis), rather than waiting for the full record to be available (which typically takes 1–2 years). This allowed our study team to recruit cancer patients shortly after diagnosis. After receiving a list of potential participants from the GBACR, letters were sent to the physician on record to ensure no medical contraindication for study participation. If there were no responses from the physician after 14 days, an invitation letter with an opt-out option was sent to the potential participant. After 14 days, recruiters called participants who did not respond to the invitation letter to assess interest and eligibility. Consent was obtained over the phone, in person, or by postal mail. For interested participants who deferred to family members, we obtained verbal consent from the participant to allow us to speak to them. Additional recruitment approaches included informing healthcare providers and community organizations about the study and distributing flyers.

The inclusion criteria were self-identify as Asian or Asian American; age 21 years or older; speak English, Cantonese, Mandarin, or Vietnamese; have stage I–III colon, rectum, liver, or lung cancer; receive healthcare in one of nine Greater Bay Area counties; were currently receiving treatment or deciding to receive treatment; and were willing to stay in the study for 6 months. The exclusion criteria were any medical or psychological conditions precluding informed consent and if the patient already completed treatment.

### Survey administration

Participants completed surveys at baseline, follow-up surveys at 3 and 6 months, and a user experience survey at 7 months. Surveys were conducted in-person, by phone, or via postal mail based on participants’ preferences. Surveys were administered in English, Chinese (Cantonese or Mandarin), or Vietnamese. Participants received a $25 gift card upon completion of each survey.

The data analyses of this study were limited to the data obtained from the surveys obtained at baseline, 3-, and 6-month follow-ups. The surveys included questions about sociodemographics, healthcare access, cancer diagnosis, staging, treatment, and quality of life. Cancer needs assessments at each time point asked participants to indicate (yes/no) to the following needs types:

1. Healthcare access
2. Cancer information: cancer diagnosis and staging, cancer treatment options, and talking with friends and family about cancer diagnosis
3. Coping with side effects: ways to cope with side effects such as fatigue, hair loss, and nausea
4. Daily living: financial matters related to cancer care, transportation, legal concerns, housing, and food access
5. Language assistance: medical interpretation and translation
6. Mental health resources: seeking help for mental health, emotions, or anxiety
7. Nutrition and physical activity
8. Substance use: information and cessation resources related to smoking, tobacco use, alcohol use, and other substance use

### Patient navigation

Participants were assigned a language-concordant patient navigator. These navigators were non-health professionals with some experience in health education and trained through the Shanti Project, a community organization with over 40 years of experience training navigators [16]. Over the course of 6 months, the navigators stayed in touch with participants via phone, in-person, email, text, or through WeChat, a popular social media application among Chinese users [17]. Based on participants’ indicated needs, navigators provided participants with an individually tailored list of local and online resources, which was compiled based on recommendations from the study’s PAC. Before the COVID-19 pandemic, navigators sometimes met participants in-person to conduct surveys and even accompanied some to their doctor visits. After March 2020, all participant contacts were completed remotely via phone call, email, text messaging, WeChat, or postal mail.

### Data analysis

Descriptive statistics were performed using SPSS v27 (IBM). Due to the small sample size (*n* = 24), we did not conduct comparison statistics by sociodemographic and clinical characteristics. Instead, we described the needs reported by participants in terms of the number of needs types they reported during the study and the proportions of participants that reported each specific needs type at each time point (baseline, 3-, and 6-month follow-ups).

## Results

**Fig. 1:**
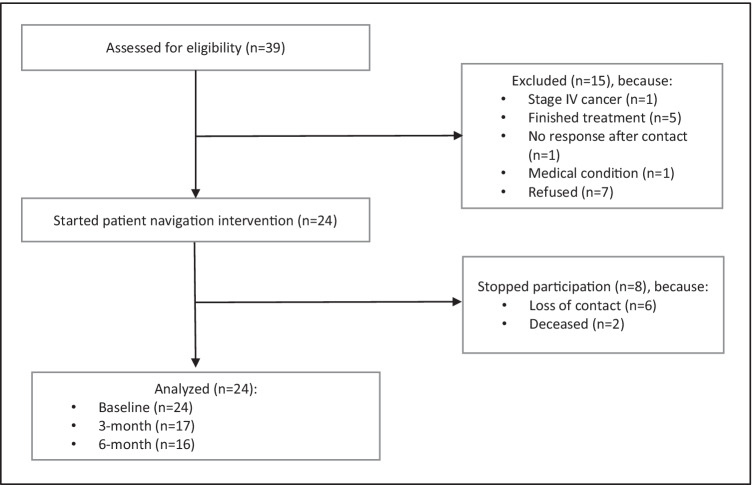
Participant flow diagram

Out of 39 potential participants assessed for eligibility, 24 were eligible and enrolled (Fig. 1). The study sample included 63% men, 58% age 65 years or older (mean age = 64.5, SD = 11.9; age range 38–81), and 42% who did not complete high school. A majority (75%) had LEP, with preferred languages being Cantonese or Mandarin (61%), Vietnamese (26%), and English (13%). Participants identified as Chinese, Filipino, or Vietnamese. A majority (92%) of participants were born outside of the USA. Most participants had stage I (54%) or III (42%) cancer of the lung (42%), colon (37%), or liver (21%). At baseline, 75% started at least one type of treatment, with 54% having had surgery and 29% chemotherapy (Table 1).

**Table 1:** Participant characteristics (*n* = 24)

	Frequency	Percentage
Gender		
Female	9	38%
Male	15	63%
Age		
Under 55	4	17%
55–64	6	25%
65–74	8	33%
75 +	6	25%
Ethnicity		
Chinese	18	75%
Other Asian*	6	25%
Preferred language		
Chinese (Cantonese or Mandarin)	16	67%
English	4	17%
Vietnamese	4	17%
English proficiency		
Very well or well	6	25%
Not well	10	42%
Not at all	8	33%
Education		
No formal education	2	8%
Elementary school	5	21%
Some high school	3	13%
High school graduate	7	29%
Attended college or above	7	29%
Employment		
Working part/full time or housemaker	8	33%
Not working due to disability, health, or other circumstances	5	21%
Unemployed and looking for employment	1	4%
Retired	10	42%
Household annual income		
< $10,000	10	42%
$10,000–20,000	6	25%
$20,000–50,000	3	13%
$50,000 +	2	8%
Refused or do not know	3	13%
Marital status		
Legally married or living together	13	54%
Separated, divorced, widowed, or single	11	46%
Cancer type		
Colorectal	9	38%
Liver	5	21%
Lung	10	42%
Cancer stage		
I or II	14	58%
III	10	42%
Cancer treatment status at baseline (categories not mutually exclusive**)**		
Had surgery	13	54%
Started chemotherapy	7	29%
Started radiation therapy	4	17%
Not started cancer treatment	6	25%

^*^Other Asian ethnicities included Filipino and Vietnamese American participants. Part of the ethnicity data was obtained from the Greater Bay Area Cancer Registry, which prohibits displaying individual ethnicity data with < 5 participants

**Fig. 2:**
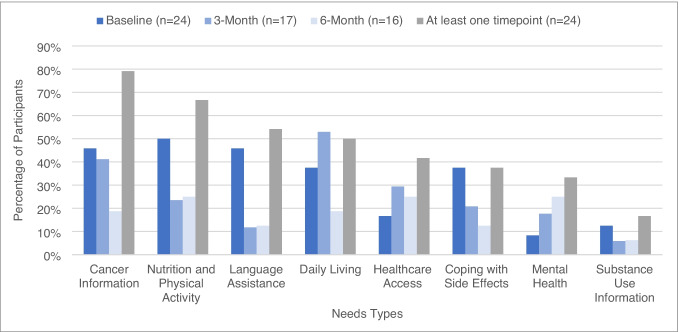
The proportion of participants that reported a needs type at baseline (*n* = 24), 3 months (*n* = 17), 6 months (*n* = 16), or at least once at any time point (*n* = 24)

Across baseline and follow-up surveys, 92% reported at least one needs type. The median number of needs types reported was 4. At least half of the participants reported the following needs types at least once in the baseline, 3-month, or 6-month surveys: cancer information (79%), nutrition and physical activity (67%), language assistance (54%), and daily living (50%) (Fig. 2).

Overall, the proportions of participants with reported needs were lower at the follow-ups than at baseline. Daily living needs were highest at 3 months compared to baseline and 6 months. However, the needs for mental health resources and healthcare access were higher at the 3- and 6-month follow-ups than at baseline (Fig. 2).

## Discussion

To our knowledge, this is among the first published studies to assess the supportive care needs of Asian American cancer patients during the course of their treatment, particularly those with LEP. Prior studies in North America, Australia, and Asia have mostly focused on Chinese cancer patients [8–11], and have not reported needs observed over different time points during cancer treatment. Other studies have focused on the needs of Asian American cancer survivors [7, 14, 18–20], but not patients who are undergoing treatment. In Patient COUNTS, we documented the needs of our participants over a 6-month period while they were undergoing cancer treatment. We observed that most participants reported at least one needs type, with three-quarters reporting three or more. For our sample that completed follow-up surveys, most needs were higher at baseline, but mental health needs were higher at 3 and 6 months.

The need for cancer information is common among cancer patients in general [21, 22]. In our study sample of cancer patients who were predominantly low-income Asian Americans with LEP, nearly 80% expressed a need for cancer and treatment information, which is consistent with other studies [8, 10]. LEP has been recognized as a barrier for Asian American patients seeking cancer supportive care [8–11]. A systematic review of the informational needs of Chinese cancer patients in Asia and North America reported that the most prevalent unmet needs are treatment information and healthcare access for immigrant Chinese patients [9]. One study using a focus group of Chinese cancer patients with LEP found that they had limited understanding of information about cancer treatment and side effects [11]. A patient navigation intervention in New York City found that the need for treatment information was among the top three needs, along with social and financial assistance, for Chinese patients with LEP [10]. Our study included Chinese, Vietnamese, and Filipino cancer participants who predominantly have LEP. Over half of our sample expressed a need for language assistance, even though our participants were receiving care in the San Francisco area. San Francisco is legally required to provide translation services in Chinese, Tagalog, and Vietnamese, which are the city’s threshold languages [4, 23, 24]. A threshold language is defined as 3000 beneficiaries or 5% of the Medi-Cal population, whose primary language is not English [24]. Our findings reveal that Asian American cancer patients need more language translation or in-language services, which should be an integral part of supportive care resources for these patients.

Financial and social support are also crucial for cancer patients [21, 22]. Studies have found that a majority of cancer patients and survivors have financial concerns, which is further associated with psychological distress [7, 25–27]. Daily living needs, including financial, legal, housing, food, and transportation, were major concerns for the predominantly low-income Asian American cancer patients in our study. Filipina American women in another study stated that for them, cancer is “one of many trials,” indicating that the struggle for daily living often supersedes even a life-threatening concern like cancer [28]. Addressing these needs is important to help Asian American cancer patients initiate and complete life-saving cancer treatments that may improve their quality of life.

At follow-ups, participants generally reported lower needs for cancer information, nutrition and physical activity, language assistance, coping with side effects, and substance use resources. One possible explanation is that over time, the needs associated with a new cancer diagnosis and treatment may have been addressed or become less relevant with appropriate cancer care [29, 30]. Another reason could be that our navigation program helped meet the needs of the participants [31].

Resources to address anxiety and depression are a common need among cancer patients [32]. Mental health and cancer are often stigmatized topics within Asian American communities [11, 14, 33–36]. One cross-sectional study of Chinese American breast cancer survivors reported that internalized stigma surrounding mental health could further compromise psychological and physical well-being of Chinese patients [14]. More participants reported having mental health needs in our 3- and 6-month follow-up surveys. One plausible explanation is that patients may focus on cancer symptoms and treatment at baseline and then later realize that they have unmet mental health needs. One study found that ethnic minority patients with lung cancer reported increased psychological needs over time during 4–12 months post-diagnosis [37]. Additionally, participants’ mental health could worsen over a 6-month period as they undergo treatment and experience side effects [38, 39]. Because two of the three cancers (liver and lung) included in Patient COUNTS tend to have worse prognosis, with rapid decline as time progresses, the patients’ mental health may have declined if their treatments were not working [37]. A third explanation could be underreporting of mental health needs due to stigmatization of mental illness [40], and after becoming more comfortable with the navigators, some participants became more open about discussing mental health needs. Lastly, some participants had baseline data collected before the COVID-19 pandemic and follow-up data collected after the pandemic started, and mental health needs rose significantly with the pandemic [41].

More participants reported having daily living needs at 3 months and healthcare access needs at 3 and 6 months. This could be due to financial toxicity, which is financial hardship due to the high cost of treatment [42] and can result in poor quality of life, emotional distress, and interrupted access to cancer care. A second explanation could be that the COVID-19 pandemic has disrupted healthcare visits and created more financial hardships for these cancer patients [43]. A third explanation could be that participants’ primary needs are in cancer information near their diagnosis at baseline, and at 3 and 6 months, their needs shift more towards daily living. Further research is warranted to address these hypotheses.

Our study has several limitations. First, we only included participants with colorectal, liver, and lung cancers, and the participants were predominantly low-income and had LEP. Thus, our findings have limited generalizability for all Asian American cancer patients. As this is a pilot project, the small sample size precluded reliable subgroup analyses to examine the associations between supportive care needs and participant characteristics. The next phase of the Patient COUNTS study will include a larger participant sample to examine supportive care needs and investigate the impacts of a novel multilingual web-based portal in providing accessible resources to Asian American cancer patients.

## Conclusion

A majority of the Asian American cancer patients in our study reported many needs. Cancer information, nutrition and physical activity, language assistance, and daily living needs are highly prevalent, highlighting the importance of providing these resources to Asian American cancer patients. We observed variation in both level and types of need over time, which suggests that these needs may be dynamic over the course of treatment and as patients transition to survivorship. The impacts from a culturally and linguistically tailored patient navigation program in addressing the cancer needs of Asian American patients should be studied further.

## Supplementary Information

Below is the link to the electronic supplementary material.

Supplementary file1 (DOCX 39 KB)
