# Supplementary material for: Patient-reported supportive care needs among Asian American cancer patients
Source: Support Care Cancer. 2022 Aug 30;30(11):9163–70. doi: 10.1007/s00520-022-07338-2 (PMC9424805; doi:10.1007/s00520-022-07338-2)
Supplement: Supplementary file 1 — Supplementary file1 (DOCX 39 KB) [file 520_2022_7338_MOESM1_ESM.docx]

**SUPPLEMENTARY FILES: NEEDS ASSESSMENT SURVEYS**

**Title:** Patient-Reported Supportive Care Needs among Asian American Cancer Patients

**Authors:** Katarina Wang^1,2^, BS; Carmen Ma^1^, BS; Feng Ming Li^1^, BS; Angeline Truong^1^, BA, MS; Salma Shariff-Marco^2,3^, PhD, MPH; Janet N Chu^1,4^, MD, MPH, MAS; Debora L Oh^2,3^, PhD, MSc; Laura Allen^2^, MPH; Mei-Chin Kuo^2^, MSN; Ching Wong^1,2,3,4^, BS; Hoan Bui^1,4^, BS; Junlin Chen^1,4^, MPH*;* Scarlett L Gomez^2,3^, PhD, MPH; Tung T Nguyen^1,3,4^, MD;  Janice Y Tsoh^1,5^, PhD

**Affiliations:**

^1^ Asian American Research Center on Health, University of California, San Francisco

^2^ Department of Epidemiology & Biostatistics, University of California, San Francisco

^3^ Helen Diller Family Comprehensive Cancer Center, University of California, San Francisco

^4^ Division of General Internal Medicine, University of California, San Francisco

^5^ Department of Psychiatry and Behavioral Sciences, University of California, San Francisco

**Corresponding Author:** Janice Tsoh; [janice.tsoh@ucsf.edu](mailto:janice.tsoh@ucsf.edu)

**Supplemental Figure 1.** Needs assessment survey (in English) that participants received at baseline. At follow-up surveys, we listed “Medical” as “Healthcare access”. At follow-up surveys, we also gave participants the option of Yes, No, and Don’t know.

# Needs Assessment (N)

N1. Please share with us, is there specific information you wish to learn more about?

[Interviewer, please make notes and allow all participants to freely describe their needs. If

helpful, you may provide some of the following as examples]

| **Information about any of these topics** | Y | Specify needs/ additional information |
| --- | --- | --- |
| 1. Cancer diagnosis and staging |  |  |
| 2. Cancer treatment options |  |  |
| 3. Communicating my conditions with friends and family |  |  |
| 4. Nutrition and physical activities |  |  |
| 5. Smoking, tobacco use |  |  |
| 6. Alcohol use |  |  |
| 7. Ways to cope with side effect: e.g., fatigue, hair loss, nausea, feeling down, feeling anxious |  |  |
| **Resources about any of these issues:** | Y | Specify needs/ additional information |
| 8. Medical |  |  |
| 9. Financial matters related cancer care |  |  |
| 10. Transportation |  |  |
| 11. Legal related matters, including employment |  |  |
| 12. Housing |  |  |
| 13. Food access |  |  |
| 14. Language, medical interpretation, translation |  |  |
| 15. Seeing a primary care doctor or general practitioner for concerns with your mental health, emotions, nerves, or use of alcohol |  |  |
| 16. Seeing a counselor, psychiatrist, psychologist, social worker or other professionals for concerns with your mental health, emotions, nerves or use of alcohol |  |  |
| 17. Other forms of assistance? please describe: |  |  |

**Supplemental Figure 2.** Needs assessment survey (in Chinese) that participants received at baseline. At follow-up surveys, we also gave participants the option of Yes, No, and Don’t know.

# Needs Assessment (N)

N1. **請與我們分享，有哪些特定的信息是您希望了解更多的嗎？**

[訪問員，請做筆記，讓所有參與者自由描述他們的需求。如果有幫助的話，您可以提供以下一些以作示例]

| 有關這些主題的信息 | 是 | 說明需求／附加的信息 |
| --- | --- | --- |
| 1. 癌症的診斷和階段 |  |  |
| 2. 癌症治療方案 |  |  |
| 3. 與朋友和家人溝通我的情況 |  |  |
| 4. 營養和體能活動 |  |  |
| 5. 吸煙，煙草的使用 |  |  |
| 6. 酒精的使用 |  |  |
| 7. 應對副作用的方法：如疲累、脫髮、噁心、情緒低落、感覺焦慮 |  |  |
| 有關這些議題的資源： | 是 | 說明需求／附加的信息 |
| 8. 醫療 |  |  |
| 9. 和癌病護理相關的財務事項 |  |  |
| 10. 交通 |  |  |
| 11.和法律相關的事宜，包括就業 |  |  |
| 12. 住屋 |  |  |
| 13. 食物供應 |  |  |
| 14. 語言、醫療解釋、翻譯 |  |  |
| 15. 為了關注您的心理健康、情緒、神經緊張或酒精的使用，去見主要的家庭醫生或全科醫生 |  |  |
| 16. 為了關注您的心理健康、情緒、神經緊張或酒精的使用，去見輔導員、精神科醫生、心理學家、社會工作者或其他專業人士 |  |  |
| 17.其他方式的援助？請描述： |  |  |

**Supplemental Figure 3.** Needs assessment survey (in Vietnamese) that participants received at baseline. At follow-up surveys, we also gave participants the option of Yes, No, and Don’t know.

Needs Assessment (N)

N1. Hãy chia sẻ với chúng tôi, có thông tin cụ thể nào bạn muốn tìm hiểu thêm không?

[Người phỏng vấn, vui lòng ghi chú và cho phép người tham gia tự do mô tả nhu cầu của họ. Nếu cảm thấy giúp gởi ý được cho họ, bạn có thể cung cấp một số thí dụ ở dước]

| **Thông tin về bất kỳ chủ đề nào** | **Y** | **Ghi rõ nhu cầu /**  **thông tin bổ sung** |
| --- | --- | --- |
| 1. Chẩn đoán và giai đoạn ung thư |  |  |
| 1. Lựa chọn cách điều trị ung thư |  |  |
| 1. Truyền đạt thông tin về tình trạng của tôi với bạn bè và gia đình |  |  |
| 4. Dinh dưỡng và hoạt động cơ thể |  |  |
| 5. Hút thuốc, sử dụng thuốc lá |  |  |
| 6. Uống rượu |  |  |
| 7. Cách đối phó với tác dụng phụ: ví dụ, mệt  mỏi, rụng tóc, buồn nôn, cảm thấy suy sụp, cảm thấy lo lắng |  |  |
| **Các nguồn tài nguyên về bất kỳ vấn đề này:** | **Y** | **Ghi rõ nhu cầu /**  **thông tin bổ sung** |
| 8. Y tế |  |  |
| 9. Vấn đề tài chính liên quan đến chăm sóc ung thư |  |  |
| 10. Giao thông di chuyển |  |  |
| 11. Các vấn đề liên quan đến pháp lý, bao gồm  cả việc làm |  |  |
| 12. Nhà ở |  |  |
| 13. Lấy được thực phẩm |  |  |
| 14. Ngôn ngữ, giải thích y học, thông dịch |  |  |
| 15. Gặp bác sĩ chăm sóc chính/gia đình hoặc bác sĩ đa khoa về sự quan tâm đến sức khỏe tâm thần, cảm xúc, thần kinh hoặc sử dụng rượu của bạn |  |  |
| 16. Gặp cố vấn, bác sĩ tâm thần, nhà tâm lý học, nhân viên xã hội hoặc các chuyên gia khác về sự quan tâm đến sức khỏe tâm thần,cảm xúc, thần kinh hoặc sử dụng rượu của bạn |  |  |
| 17. Các hình thức hỗ trợ khác? xin miêu tả: |  |  |
